# Effects of low dimensionality on electronic structure and thermoelectric properties of bismuth

**DOI:** 10.1039/c9ra08341c

**Published:** 2019-12-09

**Authors:** C. Y. Wu, L. Sun, J. C. Han, H. R. Gong

**Affiliations:** State Key Laboratory of Powder Metallurgy, Central South University Changsha Hunan 410083 China gonghr@csu.edu.cn; Department of Educational Science, Hunan First Normal University Changsha Hunan 410205 China

## Abstract

First-principles calculations and Boltzmann transport theory have been combined to comparatively investigate the band structure, phonon spectrum, lattice thermal conductivity, electronic transport properties, Seebeck coefficients, and figure of merit of the β-bismuth monolayer and bulk Bi. Calculation reveals that low dimensionality can bring about the semimetal-semiconductor transition, decrease the lattice thermal conductivity, and increase the Seebeck coefficient of Bi. The relaxation time of electrons and holes is calculated according to the deformation potential theory, and is found to be more accurate than those reported in the literature. It is also shown that compared with Bi bulk, the β-bismuth monolayer possesses much lower electrical conductivity and electric thermal conductivity, while its figure of merit seems much bigger. The derived results are in good agreement with experimental results in the literature, and could provide a deep understanding of various properties of the β-bismuth monolayer.

## Abstract 2

First-principles calculations and Boltzmann transport theory have been combined to comparatively investigate the band structure, phonon spectrum, lattice thermal conductivity, and the transport properties of the β-bismuth monolayer and bulk Bi.

## Graphical abstract

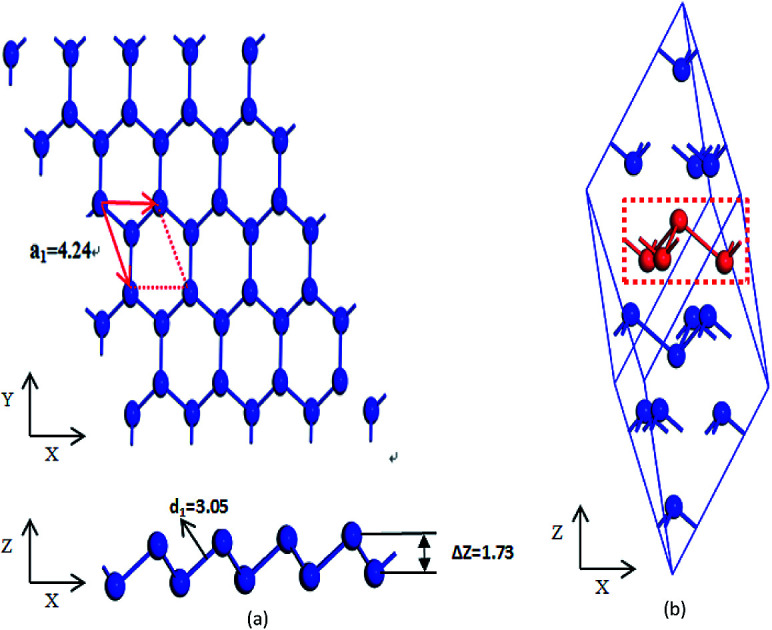

## 1. Introduction

Due to the noteworthy transport properties and potential applications for cooling and power generation, thermoelectric materials have been extensively studied during the past few decades.^1,2^ Specifically, bismuth (Bi) and its compounds have been studied for their superior thermoelectric properties at low temperatures.^3,4^ The efficiency of thermoelectric conversion can be quantified using the dimensionless thermoelectric figure of merit^5^1
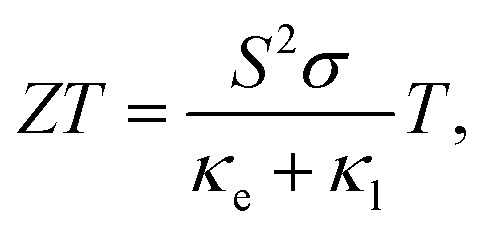
where *S* is the Seebeck coefficient, *σ* is the electrical conductivity, *T* is the absolute temperature, and *κ*_e_ and *κ*_l_ are thermal conductivity of electrons and lattice contributions, respectively. Therefore, the investigations in the literature are mainly focused on improving the *ZT* value for high conversion efficiency by achieving high *S* and *σ* values and the low thermal conductivity in the existing bulk bismuth-based compounds during the past several years.^6–8^ Unfortunately, it is extremely difficult to significantly improve their thermoelectric performance owing to the above coupled transport coefficients.

Recently, low dimensionality as one of the most promising strategies has been proposed to significantly enhance the thermoelectric performance through tuning the band structure as well as decreasing the lattice thermal conductivity.^9^ So there is an explosive growth of interest for the exploration of two-dimensional (2D) materials of the group-VA monolayers, *i.e.*, the monolayers of α-phosphorus, β-phosphorus, α-arsenic, β-arsenic, α-antimony, β-antimony, α-bismuth, and β-bismuth,^10,11^ which potentially have suitable electronic band structures for good thermoelectric properties. In particular, the β-bismuth monolayer has excellent thermoelectric properties as well as band structures, and has been well regarded as a competitive candidate in the application fields of thermoelectric components and electronic devices.^12^

Nine monolayer configurations of low-dimensional bismuth family members have been revealed, which are the honeycomb α, β, γ, δ, ε and non-honeycomb ξ, η, θ, ι nanosheets. The β-bismuth monolayer with a buckled form is the most stable structure among the low-dimensional bismuth allotropes according to the binding energy.^13^ In addition, the β-bismuth monolayer can be fabricated successfully from experiments according to the experimental cleavage energy.^14^

Interestingly, the electronic structure and thermoelectric properties of the β-bismuth monolayer have been investigated in recent years, while these results from various groups are not consistent with each other.^15–18^ For instance, the band gap of the β-bismuth monolayer covers a wide range from 0.36 to 0.99 eV according to several theoretical studies.^13^ In addition, very high *ZT* values of 2.1 and 2.4 have been reported for n-type and p-type β-bismuth monolayers at 300 K, respectively, by means of the combination of first principles, Boltzmann theory, molecular dynamics, and fitted relaxation time.^16^ On the contrary, the low *ZT* values of 0.4 and 0.60 have been predicted for n-type and p-type β-bismuth monolayers at 300 K, respectively, through *ab initio* calculation, Boltzmann theory, and a series of assumed relaxation time.^18^ Fundamentally, the β-bismuth monolayer should have an intrinsic band gap and *ZT* value, which are irrelevant to theoretical or experimental methods.

The above points imply that further theoretical studies are needed to elucidate the tremendous difference of *ZT* values of the β-bismuth monolayer owing to the different relaxation time used in the calculation. In the present study, first principle calculation and Boltzmann transport theory are therefore combined to investigate the electronic structure and thermoelectric properties of the β-bismuth monolayer. Specifically, the relaxation time is calculated by the deformation potential method^19^ and the corresponding properties of Bi bulk are also derived for the sake of comparison. The intrinsic reason of the tremendous difference of *ZT* values of the β-bismuth monolayer is clarified and the fundamental effects of low dimensionality on various properties of Bi are revealed and discussed, to provide a deep understanding of various properties of Bi.

## 2. Theoretical methods

Optimized atomic structure, phonon spectrum, and electronic structures of both β-bismuth monolayer and A7 structure of Bi are calculated by means of the well-established Vienna *ab initio* simulation package (VASP) within the density functional theory (DFT).^20,21^ The calculations are performed in a plane-wave basis with the projector-augmented wave (PAW) method.^22–24^ The local density approximation (LDA) with the inclusion of spin–orbit coupling (SOC) is employed for the exchange and correlation functions,^25,26^ which has been successfully used in electronic structure calculation of the A7 structure of Bi and bismuth monolayer in the literature.^12,27–29^

**Fig. 1:**
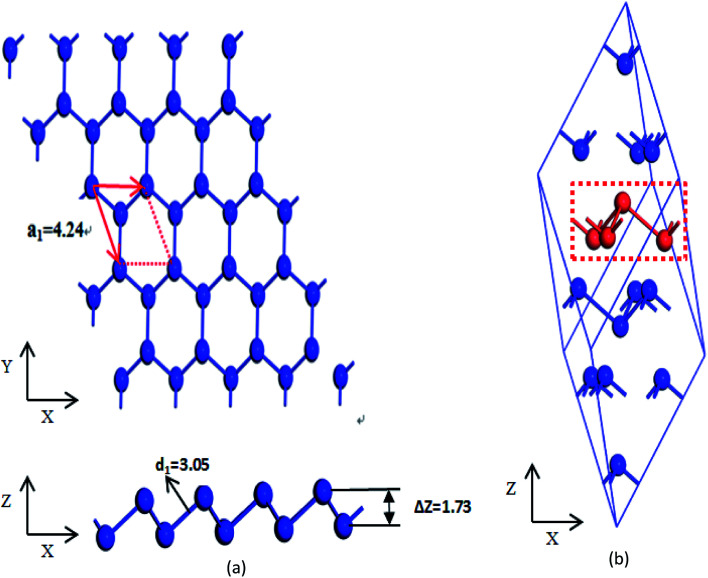
Comparison of (a) β-bismuth monolayer and (b) Bi bulk with the A7 structure.

**Fig. 2:**
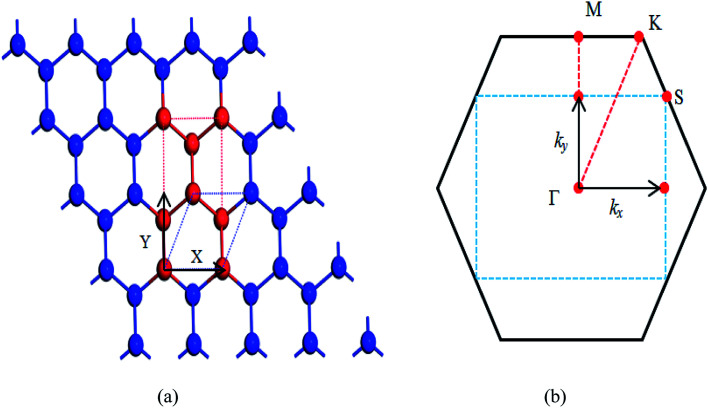
(a) Unit cell and (b) first Brillouin zones of the β-bismuth monolayer.

The β-bismuth monolayer with a rhombic lattice containing two bismuth atoms shown in Fig. 1 is used for the calculations of lattice structure, the cleavage energy, band structure, phonon spectrum, and lattice thermal conductivity. In addition, a rectangular lattice with four Bi atoms per unit cell of the β-bismuth monolayer shown in Fig. 2 is selected to calculate the electronic transport properties. After a series of test calculations, the vacuum distance is set as 15 Å to avoid the interactions between the layer and its periodic images. In addition, the k meshes of 15 × 15 × 1, 5 × 5 × 1, 15 × 9 × 1, and 35 × 21 × 1 are selected for the calculations of the lattice constant and electronic structure of a rhombic lattice, phonon spectrum of a rhombic lattice, relaxation time, and transport properties of a rectangular lattice, respectively. The energy criteria are 0.001 and 0.01 meV for electronic and ionic relaxations, respectively.

The transport properties of the β-bismuth monolayer with the rectangular lattice are derived by means of the Boltzmann transport theory and the rigid band approach (RBA) as included in the software of Boltztrap.^30^ The energy eigenvalues are employed on a very dense nonshifted 29 400 *k*-point mesh in the full Brillouin zone (BZ) from the self-consistent converged electronic structure calculations. The transport properties are derived as a function of temperature and chemical potential employing the constant relaxation time approximation (CRTA), which neglects the weak energy dependence of relaxation time (*τ*) but retains some temperature and doping dependence.^31,32^ The effects of temperature and carrier density (*n*) are simulated using the rigid band approximation,^5^ which assumes that the effects do not change the shape of the band structure, but only shift the Fermi energy.^33–35^

The lattice thermal conductivity and phonon spectrum of the β-bismuth monolayer are calculated by using the Boltzmann transport equation for the phonons as implemented in ShengBTE code^36^ and PHONOPY package.^37^ To obtain the phonon spectrum and the lattice thermal conductivity, the second-order harmonic interatomic force constants (IFCs) are calculated by using density-functional perturbation theory (DFPT) with the 5 × 5 × 1 supercell.^38^ In addition, the third-order anharmonic IFCs are performed by using the 4 × 4 × 1 supercell and the interactions up to eighth nearest neighbors are taken into account with the finite difference method for calculating lattice thermal conductivity,^39^ while the k meshes of 5 × 5 × 1 are selected for the supercell calculation with the rhombic lattice. For comparison, the phonon spectrum of the A7 structure of Bi is calculated using the 4 × 4 × 1 supercell with the finite difference method by PHONOPY package^37^ and the k meshes of 5 × 5 × 1 are adopted.

## 3. Results and discussion

### 3.1 Effects of low dimensionality on band structure of Bi

**Fig. 3:**
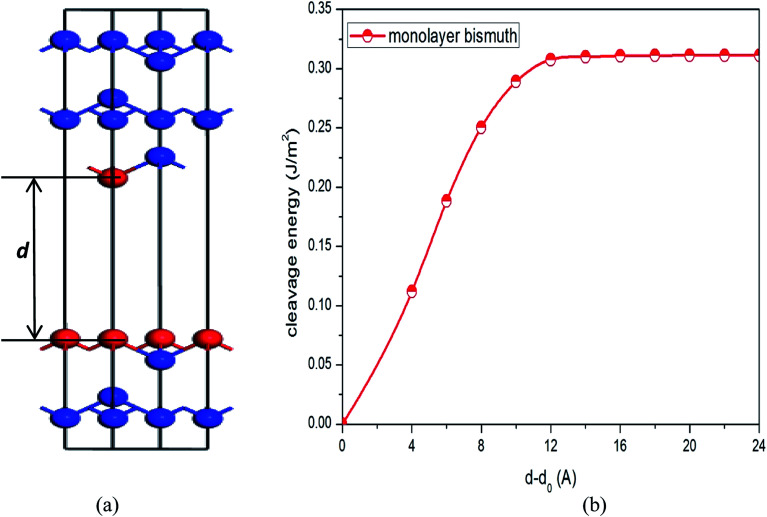
(a) Atomic model of bismuth used to simulate the exfoliation procedure and (b) corresponding cleavage energy as a function of the separation distance (*d*) between two fractured parts. *d*_0_ represents interlayer distance of equilibrium bismuth.

To simulate the exfoliation procedure of the β-bismuth monolayer from bulk Bi, the cleavage energy is calculated and shown in Fig. 3 as a function of the interlayer distance (*d*) between Bi–Bi bilayers. It can be discerned from Fig. 3b that the cleavage energy increases with the gradual increase of *d* and finally converges at the value of 0.31 J m^−2^. Such a low cleavage energy implies that it seems energetically easy to exfoliate a single layer from the A7 structure of Bi, which agrees well with similar experimental observations by Reis *et al.*^40^ Accordingly, Fig. 1 shows the obtained β-bismuth monolayer, which is a graphene-like buckled honeycomb structure with a space group of *P*3̄*m*1, and Fig. 2 displays the Brillouin zone of the β-bismuth monolayer as well as the orthorhombic supercell.

The calculated lattice constants (*a*_A7_), bond lengths (*d*_1_), buckling parameter (Δ*Z*), and unit volume (*V*) of the β-bismuth monolayer are summarized in Table 1. The available theoretical results of the β-bismuth monolayer and Bi bulk with the A7 structure in the literature^41,42^ are also included in Table 1 for comparison. It can be seen from Table 1 that the lattice constant (*a*_A7_) of the β-bismuth monolayer is less than that of Bi bulk,^43^ while its buckling parameter (Δ*Z*) seems bigger. It therefore indicates that low dimensionality has an important effect to decrease the lattice constant and increase the buckling parameter of Bi. Interestingly, the bond length (*d*_1_) and unit volume (*V*) of the β-bismuth monolayer are very close to those of Bi bulk.

**Table 1:** Optimized lattice constant *a*_A7_ (Å), bond length *d*_1_ (Å), buckling parameter Δ*Z* (Å), and unit volume (*V*) of the β-Bi monolayer and Bi bulk with the A7 structure

Structure	*a*_A7_	Δ*Z*	*d*_1_	*V* (Å^3^ per atom)	Method	Reference
β-Monolayer	4.24	1.73	3.05	22.37	LDA + SOC	This work
β-Monolayer	4.34	1.73	3.07	23.05	LDA + SOC	41
β-Monolayer	4.36	1.73	3.09	23.30	GGA + PBE + SOC	42
Bulk (A7)	4.54	1.60	3.07	22.30	LDA + SOC	44

**Fig. 4:**
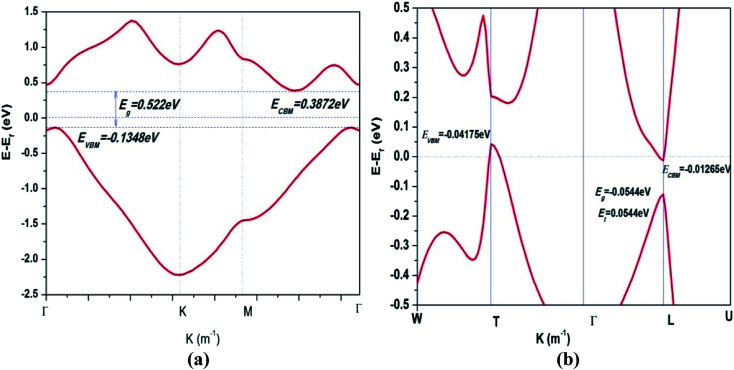
Band structures of (a) β-bismuth monolayer and (b) Bi bulk with the A7 structure.^44^

To study the effects of low dimensionality on electronic structure, the band structure of the β-bismuth monolayer is calculated and shown in Table 2 and Fig. 4. In addition, the related theoretical and experimental band structure of β-bismuth monolayer and Bi bulk with the A7 structure are also included in Table 2 and Fig. 4 for the sake of comparison. First of all, low dimensionality has an important effect to change the energy bands of Bi near the Fermi level (*E*_f_), *i.e.*, the *T* and *L* bands in Bi bulk are very close to *E*_f_ and determine the main features of its band structure, while the energy bands along the *Γ*–*M* direction in the β-bismuth monolayer are near *E*_f_ and play a decisive role.

**Table 2:** Valence band maximum (*E*_VBM_), conduction band minimum (*E*_CBM_), overlap (*E*_t_), and band gap (*E*_g_) of the β-Bi monolayer and Bi bulk with the A7 structure

Structure	*E*_VBM_ (eV)	*E*_CBM_ (eV)	*E*_t_ (eV)	*E*_g_ (eV)	Method	Reference
β-Monolayer	−0.1348	0.3872	0	0.522	LDA + SOC	This work
β-Monolayer				0.80	Experiment	40
β-Monolayer				0.51	LDA + SOC	41
β-Monolayer				0.46	GGA + PBE + SOC	17
β-Monolayer				0.50	GGA + PW91 + SOC	16
β-Monolayer				0.49	GGA + PBE + SOC	18
β-Monolayer				0.43	GGA + PBE + SOC	43
Bulk (A7)	−0.04175	−0.01265	0.0544	−0.054	LDA + SOC	44

Secondly, it can be seen from Fig. 4 that the valence band maximum (VBM) and conduction band minimum (CBM) of the β-bismuth monolayer are lower and higher than those of Bi bulk, respectively. Consequently, β-bismuth monolayer becomes a typical indirect semiconductor, whereas the original Bi bulk is a typical semimetal. In other words, low dimensionality could induce the semimetal-semiconductor (SMSC) transition of Bi, which agrees well with other results in the literature.^16–18,40–42,44^ Such a SMSC phase transition would be attributed to the quantum confinement effect through the reduction of the thickness along the *z* direction.^16^

Thirdly, the band gap (0.522 eV) of β-bismuth monolayer from the present PAW-LDA-SOC method is in good agreement with the calculated values of 0.49, 0.46, 0.50, 0.47, and 0.43 eV from other theoretical methods of GGA-PBE-SOC, GGA-PBE-SOC, GGA-PW91-SOC, GGA-PBE-SOC,^16–18,40,41,43^ respectively. It should be noted that the theoretical values of band gap of β-bismuth monolayer are all smaller than the experimental value of 0.8 eV,^40^ and such an underestimation of band gap is due to the well-known feature of density functional theory. Interestingly, the present band gap of 0.522 eV of β-bismuth monolayer seems a little bit closer to the experimental value than those from other theoretical methods.

### 3.2 Effects of low dimensionality on phonon spectrum and lattice thermal conductivity of Bi

**Fig. 5:**
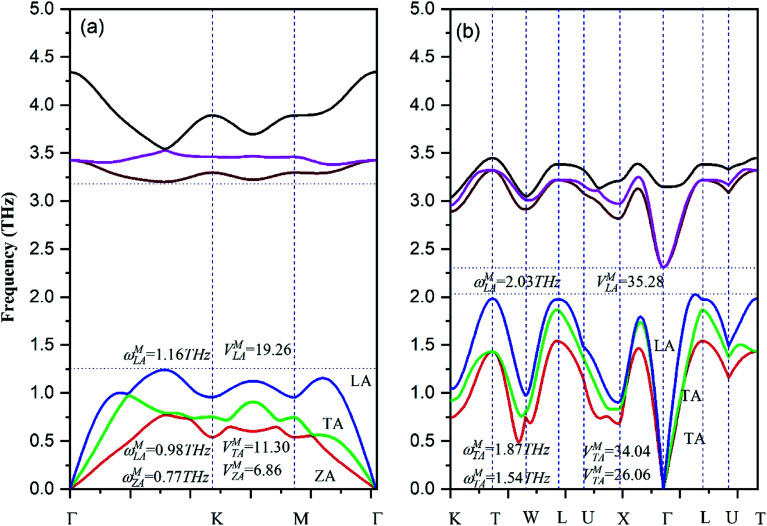
Phonon band structures of (a) β-bismuth monolayer and (b) Bi bulk with the A7 structure.

It is of importance to fundamentally understand the effects of low dimensionality on phonon transport properties. First of all, the phonon spectrums of both β-bismuth monolayer and Bi bulk with the A7 structure are calculated by the finite difference method implemented in the Phonopy package,^37^ and are shown in Fig. 5. One can discern from Fig. 5 that the phonon spectrums of the β-bismuth monolayer and Bi bulk with the A7 structure are free from imaginary frequencies in the first Brillouin zone, indicating that these two structures are thermodynamically stable, which are in good agreement with similar theoretical and experimental results in the published papers.^16,40,41^ Moreover, the longitudinal acoustic (LA) and transverse acoustic (TA) branches of both β-bismuth monolayer and Bi bulk with the A7 structure are linear in wave vector *q* close to the *Γ* point, whereas the *z*-direction acoustic (ZA) branch of β-bismuth monolayer deviates from linearity close to the *Γ* point which is a generic feature of layered material.^45^

**Fig. 6:**
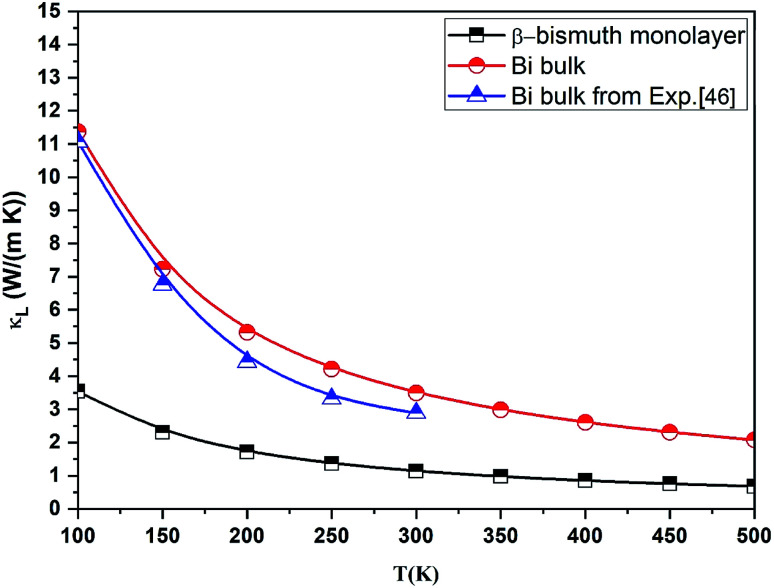
Lattice thermal conductivity (*κ*_l_) of β-bismuth monolayer and Bi bulk with the A7 structure as a function of temperature. The corresponding experimental values^46^ of Bi bulk with the A7 structure are also listed for comparison.

The lattice thermal conductivity (*κ*_l_) of β-bismuth monolayer and Bi bulk with the A7 structure is then calculated as a function of temperature in the framework of the supercell approach through using the obtained second- and third-order interatomic force constants as implemented in the ShengBTE.^36^ Consequently, Fig. 6 shows the derived lattice thermal conductivity (*κ*_l_) of β-bismuth monolayer and Bi bulk with the A7 structure as the function of temperature. The corresponding experimental values^46^ of Bi bulk with the A7 structure are also listed for comparison. One can observe from this figure that the present lattice thermal conductivity of Bi bulk is compatible with experimental values,^46^ while the calculated value has a little bit overestimation. Furthermore, low dimensionality has an important effect to decreases the lattice thermal conductivity of Bi, and the fundamental reason of such a dramatic decrease will be revealed later.

It is of interest to compare the lattice thermal conductivity of β-bismuth monolayer from the present study with those reported values in the literature. For instance, the lattice thermal conductivity of β-bismuth monolayer from the present ShengBTE code is 1.23 W m^−1^ K^−1^ at the room-temperature, which seems a little bit bigger than the theoretical value of 0.89 W m^−1^ K^−1^*via* the Phono3py code,^18^ while far less than 3.9 W m^−1^ K^−1^ from molecular dynamics (MD) simulations.^16^ According to our understanding, such a difference of the lattice thermal conductivity of β-bismuth monolayer would be mainly due to the thickness of 2D materials with a vacuum layer, *e.g.*, the thickness of the β-bismuth monolayer are 4 and 18 Å corresponding to the *κ*_l_ values of 3.9 and 0.89 W m^−1^ K^−1^*via* MD or the Pono3py code,^16,18^ respectively, while the tested thickness of the β-bismuth monolayer in our work is 31.73 Å, in order to obtain reliable lattice thermal conductivity.

We now turn to reveal the intrinsic reason why low dimensionality can decrease the lattice thermal conductivity of Bi in terms of phonon band structures shown in Fig. 5 and the three-phonon processes. Accordingly, the lattice thermal of conductivity of β-bismuth monolayer and Bi bulk with the A7 structure could be obtained from the summation of contribution of all the phonon modes by the phonon kinetic theory as follows:^47^2
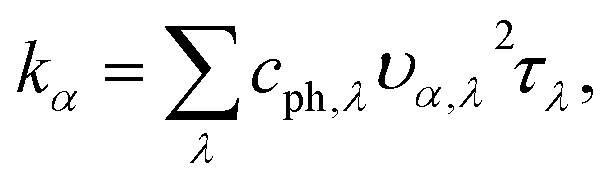
where *κ*_*α*_, *c*_ph,*λ*_, *υ*_*α*,*λ*_, and *τ*_*λ*_ are the lattice thermal conductivity in *α* direction, the phonon volumetric specific heat of mode *λ*, the phonon group velocity of mode *λ* along *α* direction, and the phonon lifetime of mode *λ*, respectively. Accordingly, the group velocity could be defined as:^48^3
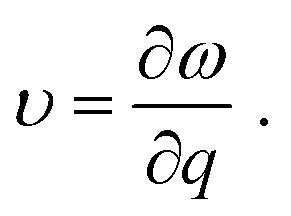


It can be seen clearly from Fig. 5 that the maximum frequencies of ZA, TA, and LA of the β-Bi monolayer are 0.77, 0.98, and 1.16 THz, respectively, which are smaller than the corresponding values of 1.54, 1.87, and 2.03 THz of Bi bulk with the A7 structure. Similarly, the maximum group velocity of the β-Bi monolayer are 1.926, 1.130, and 0.686 km s^−1^, respectively, lower than the corresponding values of 2.606, 3.404, and 3.528 km s^−1^ of Bi bulk with the A7 structure. The above comparisons suggest that lower phonon velocity and acoustic-phonon frequency lead to the lower lattice thermal conductivity of the β-Bi monolayer.

**Fig. 7:**
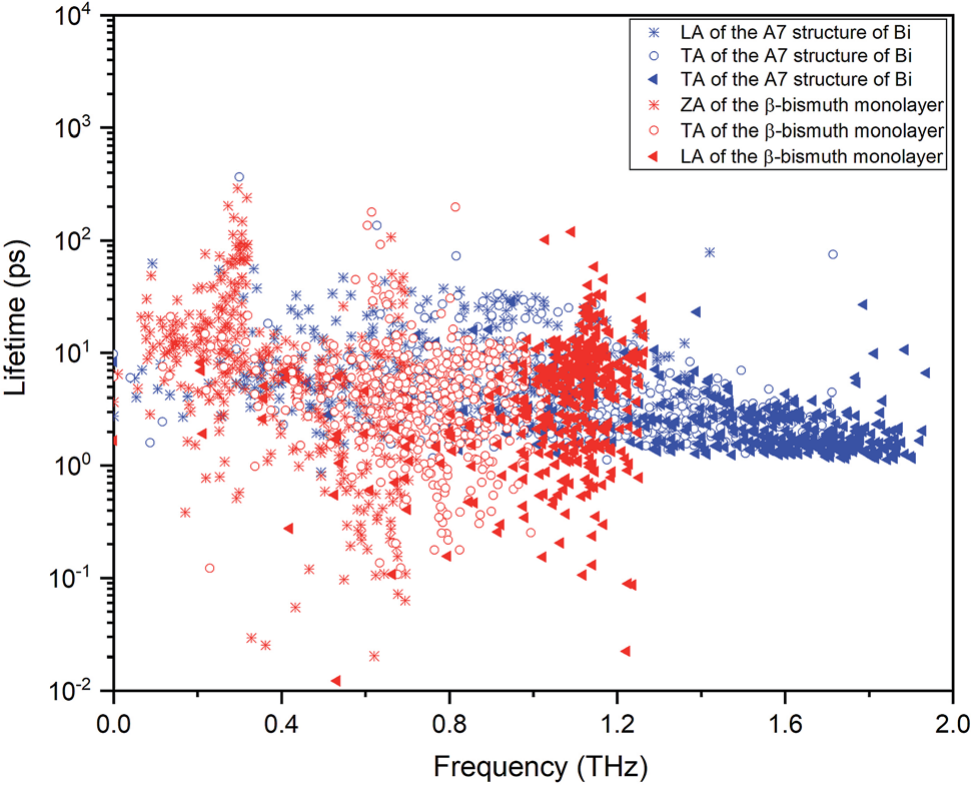
The lifetimes of the phonon mode of β-Bi monolayer and the A7 structure of Bi as the function of the frequency.

To find out the effect of the phonon lifetime on the lattice thermal conductivity, Fig. 7 lists the phonon mode lifetimes of β-Bi monolayer and the A7 structure of Bi as the function of the frequency. One could see that the phonon lifetime of the β-Bi monolayer is slightly lower than the A7 structure of Bi, implying that the lower phonon lifetime of the β-Bi monolayer leads to the lower lattice thermal conductivity than that of the A7 structure of Bi. It should be noted that the influence of the phonon group velocity and phonon lifetime on the lattice thermal conductivity from the present study match well with similar theoretical conclusions about the 2D V monolayer family in the literature.^18,49^

### 3.3 Effects of low dimensionality on electronic transport properties and figure of merit of Bi

To find out the electrical transport properties and figure of merit of both β-bismuth monolayer and Bi bulk with the A7 structure, the relaxation time of the carriers (electrons and holes) should be calculated beforehand. In this respect, the values of relaxation time are simply assumed or approximated from the simple model in the literature.^16,18^ In the present study, the deformation potential theory (DP)^50,51^ is performed to calculate the relaxation time of Bi. It should be noted that the matrix elements of interactions between electrons and longitudinal acoustic phonons are considered in the DP theory, and the relaxation time from the DP theory would be more reliable than those assumed or approximated values in the literature.^16,18^

According to the DP theory, the relaxation time (*τ*_*i*_) along a certain direction *i* (*i* = *x*, *y*, *z*) can be derived by the following form:^12,52–54^4
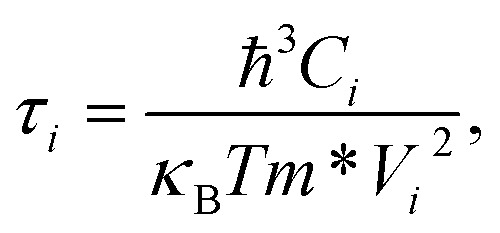
where m* is the effective masses of electrons or holes, *C*_*i*_ and *V*_*i*_ are the elastic constant and deformation potential constant along a certain direction *i* (*i* = *x*, *y*, *z*), respectively. The values of m*, *C*_*i*_, and *V*_*i*_ are calculated as follows:5
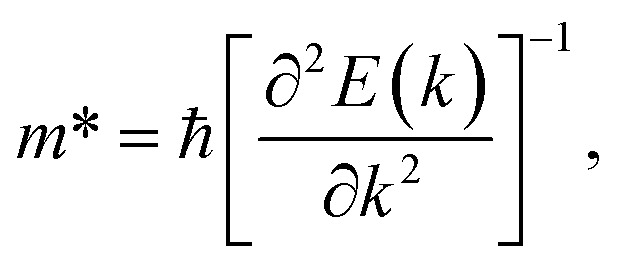
6
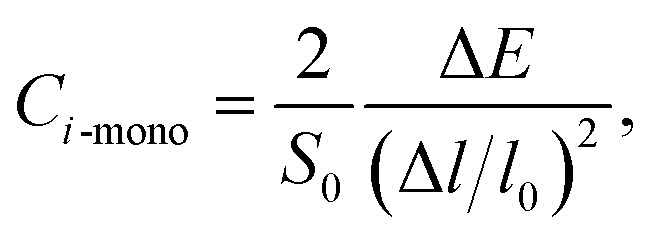
7
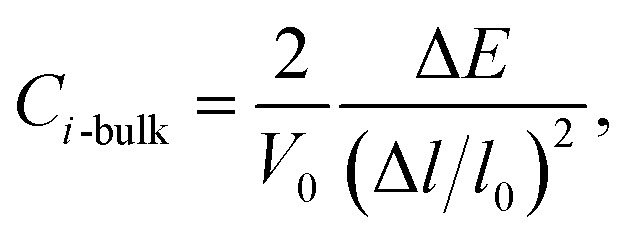
8
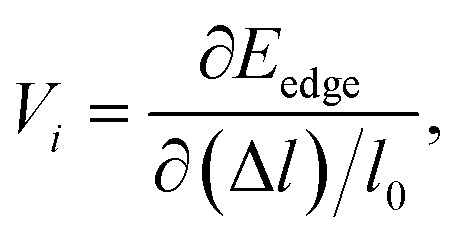
where *C*_*i*-mono_ and *C*_*i*-bulk_ are deformation potential constants of β-bismuth monolayer and Bi bulk with the A7 structure, respectively; Δ*E* is the total energy change due to the dilation of Δ*l*/*l*_0_, in which *l*_0_ is the equilibrium lattice spacing along the direction *i*, and Δ*l* = *l* − *l*_0_ is the change of the lattice spacing; *S*_0_ is the surface area of the β-bismuth monolayer, and *V*_0_ is the equilibrium volume of Bi bulk; *E*_edge_ is the valence band top or conduction band bottom, and *V*_*i*_ is the deformation potential constant which represents the shift of band edge under per unit strain.

After a series of calculations, the relaxation time of Bi is derived as a function of temperature. As a typical example, Table 3 shows the obtained elastic constants *C*_*i*_, deformation potential constant *V*_*i*_, and relaxation time *τ*_*i*_ along a certain direction *i* (*i* = *x*, *y*, *z*) of the β-Bi monolayer and Bi bulk with the A7 structure at 300 K. The effective masses m* are also listed for comparison. It should be pointed out that the *x* and *y* directions of Bi are set in Fig. 2a, and the *z* direction of Bi bulk is perpendicular to the *xy* plane. One can see clearly from Fig. 3 that the elastic constants of the β-bismuth monolayer is much lower than the corresponding values of Bi bulk, and are in good agreement with the calculated results in the literature.^41^ In addition, the relaxation time of electrons or holes in the β-bismuth monolayer along a certain direction is smaller than that in the Bi bulk, while its effective mass seems bigger.

**Table 3:** Elastic constants *C*_*i*_, deformation potential constant *V*_*i*_, and relaxation time *τ*_*i*_ along a certain direction *i* (*i* = *x*, *y*, *z*) of the β-Bi monolayer and Bi bulk with the A7 structure at 300 K. The effective masses m* are also listed for comparison. e and h represent electron and hole, respectively

	A7	β-Bi	Ref.		A7	β-Bi
*V* ^e^_*x*_ (eV)	3.52	3.08		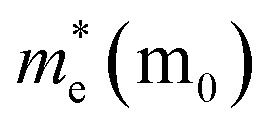	0.051	0.143
*V* ^e^_*y*_ (eV)	2.64	3.49		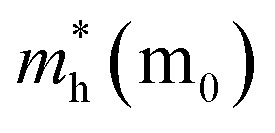	0.180	0.231
*V* ^e^_*z*_ (eV)	2.37			*τ* ^e^_*x*_ (ps)	1.09	0.44
*V* ^h^_*x*_ (eV)	4.21	1.61		*τ* ^e^_*y*_ (ps)	1.42	0.39
*V* ^h^_*y*_ (eV)	3.94	9.14		τ^e^_*z*_ (ps)	0.99	
*V* ^h^_*z*_ (eV)	1.29			*τ* ^h^_*x*_ (ps)	0.77	0.52
*C*_*x*_	72.45 GPa	23.86 N m^−1^	23.90 N m^−1^ (ref. 41)	*τ* ^h^_*y*_ (ps)	0.81	0.09
*C*_*y*_	70.64 GPa	23.96 N m^−1^	23.90 N m^−1^ (ref. 41)	*τ* ^h^_*z*_ (ps)	1.54	
*C*_*z*_	44.25 GPa					

It is of importance to compare the present relaxation time of the β-bismuth monolayer with those available in the literature.^16,18^ As shown in Table 3, for both electrons and holes, the present relaxation time along the *x* direction is different from that along the *y* direction, suggesting that the relaxation time of both electrons and holes of the β-bismuth monolayer should be anisotropic. The corresponding relaxation time of the β-bismuth monolayer in the literature, however, is assumed to be isotropic with the value of 0.148 ps (ref. 16) or 0.001, 0.01, 0.1 ps,^18^ respectively. It should be noted that the value of 0.148 ps is approximated from the experimental electronic conductivity of Bi bulk,^16^ while 0.001, 0.01, and 0.1 ps are simply estimated values.^18^ Consequently, the relaxation time of the β-bismuth monolayer from the present DP theory shown in Table 3 should be more accurate than those values in the literature.^16,18^

**Fig. 8:**
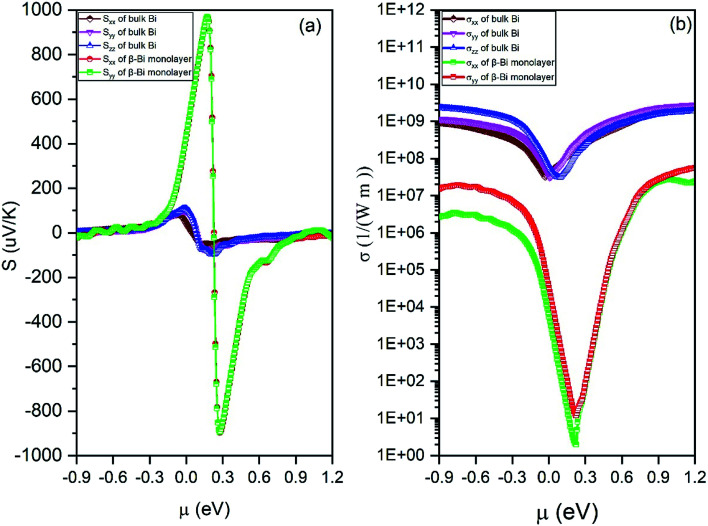
(a) Seebeck coefficients and (b) electric conductivity of β-bismuth monolayer and Bi bulk with the A7 structure at 300 K as the function of chemical potential (*μ*).

**Fig. 9:**
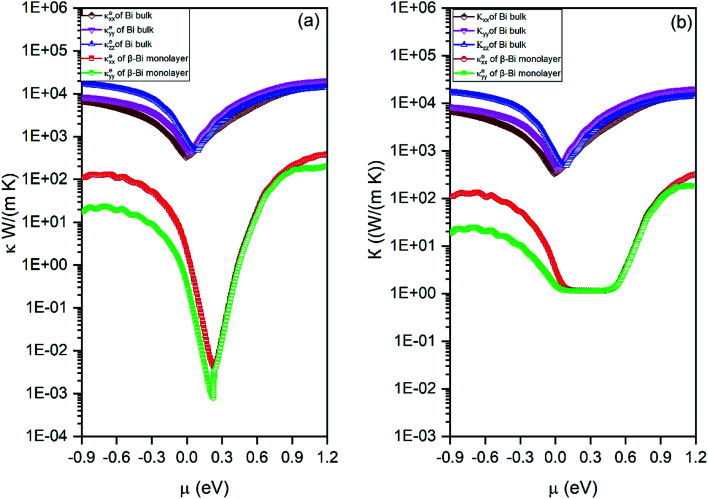
(a) Electronic thermal conductivity and (b) total thermal conductivity of β-bismuth monolayer and Bi bulk with the A7 structure at 300 K as the function of chemical potential (*μ*).

**Fig. 10:**
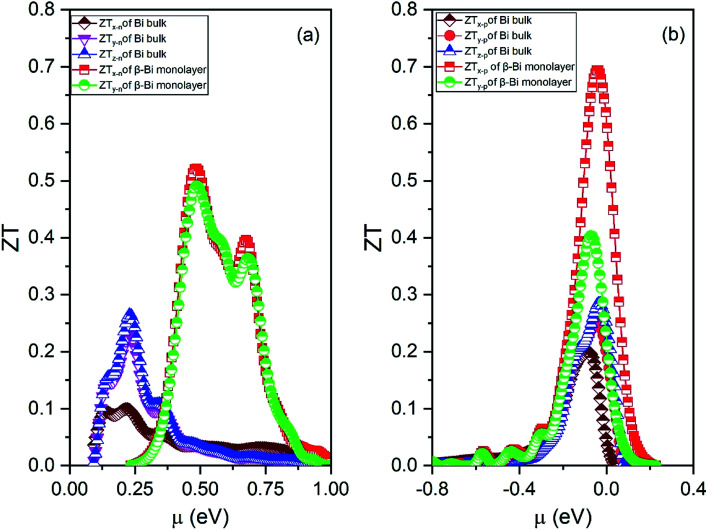
Figure of merit (*ZT*) of (a) electrons and (b) holes of β-bismuth monolayer and Bi bulk with the A7 structure at 300 K as the function of chemical potential (*μ*).

By means of the obtained relaxation time, the electrical conductivity (*σ*), electric thermal conductivity (*κ*_e_), and total thermal conductivity (*Κ* = *κ*_e_ + *κ*_L_) are calculated for both β-bismuth monolayer and Bi bulk with the A7 structure. In addition, the Seebeck coefficients (*S*) are also derived as a function of chemical potential. With the above obtained physical properties, the figure of merit of both β-bismuth monolayer and Bi bulk with the A7 structure is calculated according to eqn (1). As typical examples, Fig. 8–10 display the Seebeck coefficients, electrical conductivity (*σ*), electric thermal conductivity (*κ*_e_), total thermal conductivity (*Κ*), and figure of merit (*ZT*) of β-bismuth monolayer and Bi bulk with the A7 structure at 300 K as the function of chemical potential (*μ*).

Several characteristics could be discerned from Figs. 8–10. First of all, the absolute values of the maximum Seebeck coefficients of the β-bismuth monolayer are 898 and 967 μV K^−1^ for electrons and holes, respectively, which seem tremendously bigger than the corresponding values of 95.7 and 119.4 μV K^−1^ of Bi bulk with the A7 structure. This dramatic comparison suggests that low dimensionality should have a significant effect to increase the Seebeck coefficients of β-bismuth. Such an effect is consistent with the above-mentioned semimetal-semiconductor transition, and would be probably due to the lower carrier density of the monolayer.^44^

Secondly, the electrical conductivity (*σ*), electric thermal conductivity (*κ*_e_), and total thermal conductivity (*Κ*) of the β-bismuth monolayer are considerably lower than those of Bi bulk with the A7 structure, respectively. That is to say, low dimensionality has an important to decrease electrical conductivity and electric thermal conductivity of Bi. The shape of the curve of electronic thermal conductivity in Fig. 9 is very similar to that of the electronic conductivity in Fig. 8, which is consistent with the Wiedemann–Franz law.^55^

Thirdly, the *ZT* values of the holes and electrons of the β-bismuth monolayer along the *x*/*y* directions are all much bigger than the corresponding values of Bi bulk with the A7 structure, respectively. It therefore follows that low dimensionality could considerably improve the efficiency of thermoelectric properties of Bi, which is in good agreement with similar experimental and theoretical results in the literature.^12,16,18,56^ The maximum *ZT* value (0.69) of the holes of the β-bismuth monolayer is bigger than that (0.52) of the electrons, and such a higher *ZT* values would be attributed to the weak coupling of electrons and phonons.^12^ It should be pointed out that the maximum figure of merit (0.69) of β-bismuth monolayer from the present study is bigger than the corresponding values of 0.626 and 0.452 of β-As and β-Sb monolayers reported in the literature.^17^

It is of interest to discuss a little bit more about the figure of merit of the β-bismuth monolayer. As related before, a controversy about the *ZT* values of the β-bismuth monolayer has appeared in the literature, *i.e.*, the *ZT* values of 2.1 or 0.4 and 2.4 or 0.6 have been reported for n-type and p-type β-bismuth monolayers at 300 K, respectively,^16^ which are also different from the present values of 0.52 and 0.69. Such a dramatic difference of *ZT* values, according to our understanding, is mainly due to the different relaxation time used in the calculation. The relaxation time of 0.148 ps or 0.001, 0.01, 0.1 ps in the literature^16,18^ is estimated or approximated value, while the present relaxation time from the deformation potential theory^50,51^ should be more accurate. Consequently, the *ZT* values of the β-bismuth monolayer from the present study would be more realistic than those reported in the literature.^16,18^

## 4. Conclusions

In summary, highly accurate first principles calculation and Boltzmann transport theory have been used to find out the fundamental influence of low dimensionality on electronic structures, phonon band structures, and thermoelectric properties of Bi. Results show that low dimensionality should considerably decrease the lattice thermal conductivity, electrical conductivity, and electric thermal conductivity as a result of the semimetal–semiconductor transition. In addition, the temperature dependent relaxation time of the β-bismuth monolayer is calculated and is found to be smaller than that of Bi bulk with the A7 structure. The maximum values of figure of merit for the electrons and holes of the β-bismuth monolayer are 0.52 and 0.69, respectively, which should be much bigger than those of Bi bulk, and could be more accurate than those reported values in the literature.

## Funding

This work was supported by State Key Laboratory of Powder Metallurgy of Central South University of China, Huxiang Youth Talent Project of Hunan Province (Grant no. 2018RS3099).

## Conflicts of interest

The authors declare that they have no conflict of interest.

## Supplementary Material

## References

[cit1] Chen G., Dresselhaus M. S., Dresselhaus G., Fleurial J. P., Caillat T. (2003). Int. Mater. Rev..

[cit2] Tan G., Zhao L. D., Kanatzidis M. G. (2016). Chem. Rev..

[cit3] Issi J. P. (1979). Aust. J. Phys..

[cit4] Jin H., Jaworski C. M., Heremans J. P. (2012). Appl. Phys. Lett..

[cit5] Ong K. P., Singh D. J., Wu P. (2011). Phys. Rev. B: Condens. Matter Mater. Phys..

[cit6] Pei Y., Wang H., Snyder G. J. (2012). Adv. Mater..

[cit7] Shanyu W., Han L., Ruiming L., Gang Z., Xinfeng T. (2013). Nanotechnology.

[cit8] Ovsyannikov S. V., Morozova N. V., Korobeinikov I. V., Lukyanova L. N., Manakov A. Y., Likhacheva A. Y., Ancharov A. I., Vokhmyanin A. P., Berger I. F., Usov O. A., Kutasov V. A., Kulbachinskii V. A., Okada T., Shchennikov V. V. (2015). Appl. Phys. Lett..

[cit9] Zhou Y., Zhao L. D. (2017). Adv. Mater..

[cit10] Pumera M., Sofer Z. (2017). Adv. Mater..

[cit11] Zhang S., Xie M., Li F., Yan Z., Li Y., Kan E., Liu W., Chen Z., Zeng H. (2016). Angew. Chem., Int. Ed..

[cit12] Cheng L., Liu H. J., Zhang J., Wei J., Liang J. H., Jiang P. H., Fan D. D., Sun L., Shi J. (2016). Phys. Chem. Chem. Phys..

[cit13] Zhang S., Xie M., Li F., Yan Z., Li Y., Kan E., Liu W., Chen Z., Zeng H. (2015). Angew. Chem..

[cit14] Zacharia R., Ulbricht H., Hertel T. (2004). Phys. Rev. B: Condens. Matter Mater. Phys..

[cit15] Zhang S., Guo S., Chen Z., Wang Y., Gao H., Gómez-Herrero J., Ares P., Zamora F., Zhu Z., Zeng H. (2018). Chem. Soc. Rev..

[cit16] Cheng L., Liu H., Tan X., Zhang J., Wei J., Lv H., Shi J., Tang X. (2014). J. Phys. Chem. C.

[cit17] Liu M. Y., Huang Y., Chen Q. Y., Li Z. Y., Cao C., He Y. (2017). RSC Adv..

[cit18] Zhang D. C., Zhang A. X., Guo S. D., Duan Y. F. (2017). RSC Adv..

[cit19] Xi J., Long M., Tang L., Wang D., Shuai Z. (2012). Nanoscale.

[cit20] Kresse G., Furthmüller J. (1996). Phys. Rev. B: Condens. Matter Mater. Phys..

[cit21] Kresse G., Furthmüller J. (1996). Comput. Mater. Sci..

[cit22] Kresse G., Joubert D. (1999). Phys. Rev. B: Condens. Matter Mater. Phys..

[cit23] Liu L. C., Gong H. R., Zhou S. F., Gong X. (2019). J. Membr. Sci..

[cit24] Liu L. C., Gong H. R., Zhou S. F. (2019). Int. J. Hydrogen Energy.

[cit25] Ceperley D. M., Alder B. J. (1980). Phys. Rev. Lett..

[cit26] Gonze X., Michenaud J. P., Vigneron J. P. (1988). Phys. Scr..

[cit27] Shick A. B., Ketterson J. B., Novikov D. L., Freeman A. J. (1999). Phys. Rev. B: Condens. Matter Mater. Phys..

[cit28] Zouhar M., Šob M. (2016). Phys. Rev. B.

[cit29] Wu C. Y., Sun L., Liang C. P., Gong H. R., Chang M. L., Chen D. C. (2019). J. Phys. Chem. Solids.

[cit30] Madsen G. K. H., Singh D. J. (2006). Comput. Phys. Commun..

[cit31] Parker D., Singh D. J. (2011). Phys. Rev. X.

[cit32] Xi L., Zhang Y. B., Shi X. Y., Yang J., Shi X., Chen L. D., Zhang W., Yang J., Singh D. J. (2012). Phys. Rev. B: Condens. Matter Mater. Phys..

[cit33] Han J. C., Wu C. Y., Sun L., Gong H. R., Gong X. (2019). J. Phys. Chem. Solids.

[cit34] Wu C. Y., Sun L., Gong H. R., Zhou S. F. (2019). J. Mater. Sci..

[cit35] Sun L., Wu C. Y., Han J. C., Gong H. R., Chang M. L., Chen D. C. (2019). J. Appl. Phys..

[cit36] Li W., Carrete J., Katcho N. A., Mingo N. (2014). Comput. Phys. Commun..

[cit37] Togo A., Oba F., Tanaka I. (2008). Phys. Rev. B: Condens. Matter Mater. Phys..

[cit38] Baroni S., de Gironcoli S., Dal Corso A., Giannozzi P. (2001). Rev. Mod. Phys..

[cit39] Li W., Lindsay L., Broido D. A., Stewart D. A., Mingo N. (2012). Phys. Rev. B: Condens. Matter Mater. Phys..

[cit40] Reis F., Li G., Dudy L., Bauernfeind M., Glass S., Hanke W., Thomale R., Schäfer J., Claessen R. (2017). Science.

[cit41] Aktürk E., Aktürk O. Ü., Ciraci S. (2016). Phys. Rev. B.

[cit42] Guo Y., Pan F., Ye M., Sun X., Wang Y., Li J., Zhang X., Zhang H., Pan Y., Song Z., Yang J., Lu J. (2017). ACS Appl. Mater. Interfaces.

[cit43] Lee J., Tian W. C., Wang W. L., Yao D. X. (2015). Sci. Rep..

[cit44] Wu C. Y., Han J. C., Sun L., Gong H. R., Liang C. P. (2018). J. Phys.: Condens. Matter.

[cit45] Hartmut Z. (2001). J. Phys.: Condens. Matter.

[cit46] Gallo C. F., Chandrasekhar B. S., Sutter P. H. (1963). J. Appl. Phys..

[cit47] Yuan K., Zhang X., Li L., Tang D. (2018). Phys. Chem. Chem. Phys..

[cit48] Gao Z., Tao F., Ren J. (2018). Nanoscale.

[cit49] Guo S. D., Liu J. T. (2017). Phys. Chem. Chem. Phys..

[cit50] Bardeen J., Shockley W. (1950). Phys. Rev..

[cit51] Xi J., Long M., Tang L., Wang D., Shuai Z. (2012). Nanoscale.

[cit52] Sharma S., Kumar S., Schwingenschlögl U. (2017). Phys. Rev. Appl..

[cit53] Naghavi S. S., He J., Xia Y., Wolverton C. (2018). Chem. Mater..

[cit54] Chen J., Wang D., Shuai Z. (2012). J. Chem. Theory Comput..

[cit55] Snyder G. J. and Toberer E. S., in Materials for Sustainable Energy, Co-Published with Macmillan Publishers Ltd, UK, 2010, pp. 101–110, 10.1142/9789814317665_0016

[cit56] Hicks L. D., Dresselhaus M. S. (1993). Phys. Rev. B: Condens. Matter Mater. Phys..

